# The Prevalence of Cardiovascular Disease Risk Factors among Employees in the Kingdom of Bahrain between October 2010 and March 2011: A Cross-Sectional Study from a Workplace Health Campaign

**DOI:** 10.1155/2014/832421

**Published:** 2014-07-06

**Authors:** Ameera Ali AL-Nooh, Abdulhussain Abdulabbas Abdulla Alajmi, David Wood

**Affiliations:** ^1^Consultant Family Physician-, NCD Unit-, Public Health Directorate, Ministry of Health, Manama, Bahrain; ^2^Department of Cardiovascular Medicine (NHLI), Imperial College London, South Kensington Campus, London SW7 2AZ, UK; ^3^Consultant Family Physician/Public Health Directorate, Head of Noncommunicable Disease Unit, Ministry of Health, Manama, Bahrain; ^4^The International Center for Circulatory Health, National Heart and Lung Institute, Imperial College London, St. Mary's Hospital, Praed Street, London W2 1NY, UK; ^5^Imperial College Healthcare NHS Trust, St. Mary's Hospital, Praed Street, London W2 1NY, UK

## Abstract

*Background*. High prevalence of CVD risk factors has been reported in Bahrain.* Objective*. This study aims to estimate the CVD risk factors prevalence among government employees in Bahrain.* Design*. A cross-sectional study design.* Setting*. Different government workplaces in Bahrain.* Method*. Data was collected from 1139 employees between October 2010 and March 2011 through interviews, including physical measurements, patient blood testing, and expired carbon monoxide (CO) levels as particles per million (ppm) for smokers. A summary of composite CVD risk factors was identified.* Results*. The following overall prevalence rates were reported: overweight and obesity 78.4% and reported hypertension 36.9% (included both those who were on and not on treatments), with an estimated prevalence of 21.6% for measured systolic blood pressure (Sbp) ≥ 140 mmHg and 23.3% for diastolic blood pressure (dbp) ≥90 mmHg. The prevalence of total cholesterol levels ≥5.2 mmol/dl was 24.2% and LDL levels >3.3 mmol/dl 10.8%. Prevalence of HDL-C levels (≤1.03 mmol/dl) was 47.55% and (≥1.5 mmol/dl) in 12.31%. The low HDL level (<1.03 mmol/dl) among males was 64.1%, while it was 26.6% among females. Half the participants (50.8%) do not engage in any type of physical activity. Moreover, 24.3% were not eating daily servings of fruits and vegetables. About 16.1% of them were current smokers. The majority of the participants (95.35%) had either no or less than 3 CVD risk factors. Only 4.65% had 3–5 risk factors.* Conclusions*. Among the employees in Bahrain, the high CVD risk factors prevalence is evident. CVD risk factors prevention and control are a priority.

## 1. Introduction

Cardiovascular disease (CVD) is the leading cause of death among the noncommunicable diseases (NCDs). By reducing the prevalence of behavioral (CVD) risk factors (tobacco use, unhealthy diet, physical inactivity, and harmful use of alcohol), a large percentage of CVDs and other NCDs can be prevented [1]. NCDs are major causes of morbidity and mortality in Bahrain. Out of the 2388 total deaths reported in 2010, 379 (16%) were due to circulatory diseases [2].

According to the National NCD Risk Factors Survey in 2007, the prevalence of diabetes mellitus (DM) was 14.3%, hypertension (HTN) was 38.2%, and high cholesterol level was 40.6%. In addition, the overall prevalence of obesity was 36.3% and 32.9% overweight. Furthermore, 62% reported daily intake of vegetables, while only 49.6% reported daily intake of fruits. Around 57.1% reported that they do not engage in any physical activity at leisure time. Finally, the overall prevalence of smoking was found to be 19.9% (AL Sayyad J., MOH, Bahrain, 2010).

The majority of the epidemiological studies from the region were descriptive and among samples of subjects or volunteers from the general population with little emphasis on the workers in their workplaces. The employees in any community represent the main workforce and their quality of life, health awareness, and adoption of healthy behaviors reflect on the overall productivity, economic growth, and the disease burden. Regardless of the study sample, the overall prevalence of CVD risk factors is high and primary and secondary preventions and interventions are vital.

In a cross-sectional study done in Iran about CVD risk factors, the overall prevalence of overweight among men was 36.6% and 35.9% among women. 11.2% of men and 28.1% of women were obese. The mean BMI, waist circumference (WC), and waist hip ratio increased with age up to 65 years. The total serum cholesterol (TC), triglycerides (TG), and 2-hour postload plasma glucose increased with BMI and WC in both sexes [3]. Another cross-sectional study which was conducted in Kuwait to assess the prevalence of major CVD risk factors showed obesity prevalence of 44% (Obesity was defined as a body mass index (BMI) of 30 kilogram per square meters or greater). About 38% had TC of more than the acceptable levels [4]. A cross-sectional community based study on established CVD risk factors carried in AL-Ain City, UAE, using Framingham risk scores for risk assessment, found 28.4% to have a score of >20%, 23.3% had DM, 20.8% HTN, 37.3% obesity, 22.7% metabolic syndrome, and 19.6% of male smoked. Coronary heart disease was reported in 2.4%. Lipid profile was abnormal in 64% of the males, and in 53% of the females, mostly due to LDL, or high TG level [5].

Weqaya is a population-wide CVD screening program in Abu Dhabi, UAE, in which self-reported indicators, anthropometric measures, and blood tests were used to screen 138 adults aged 18 years or older. Participants mean age was 36.82 years; 43% were men. Risk factors prevalence rates were as follows: obesity 35%, overweight 32%, central obesity 55%, DM 18%, pre-DM 27%, dyslipidemia 44%, and HTN 23.1%. In addition, 26% of men were smokers, compared with 0.8% of women. Age standardized DM and pre-DM rates were 25% and 30%, respectively, and age standardized rates of obesity and overweight were 41% and 34% [6].

A study was conducted to determine the prevalence of CHD risk factors among 159 male university students in Dammam City, Saudi Arabia. It was found that 28.9% of the University students do not practice any type of physical activity. Moreover, 37.7% and 46.5% of them were watching TV and using computer for 14 hours and more, respectively. About 19% of them were current smokers. 24.5% were overweight, 11.9% were obese, and 10.7% were severely obese based on BMI and waist-hip ratio. The mean systolic and diastolic blood pressure readings of students were 122.8 mmHg and 71.5 mmHg, respectively [7].

The research into the epidemiology of physical activity in Saudi Arabia and neighboring countries is sparse. From a brief review of published data about the level of physical activity in Saudi Arabia from 1990 to 2004, around 8 studies were found. Across the studies, the total rate of inactivity ranged from 43.3% to 99.5%. Only 2 of the studies included data for both males and females. Their data indicated that females were much less active than males [8].

The high prevalence of CVD in the region seems to be communal. This descriptive study will determine the prevalence in the Kingdom and provides a base for future descriptive and analytical studies which will aid in planning and provision of primary and secondary CVD prevention services and population based health promotion programs. Studying CVD risk factors among the workers will lead to recommendations that can motivate employers and policy makers to put CVD prevention among their priorities. In addition, it will enforce legislations in tobacco control and food labeling and help in promotional activities for the employees.

## 2. The Methods

### 2.1. Study Design and Justification

A cross-sectional work-site based survey was used to describe the prevalence of some of CVD risk factors among government employees in Bahrain.

### 2.2. Indication and Justification of the Sample Size

The sample size was calculated to ensure representation of the population and was estimated to be 1139 employees as the total number of the government employees was unknown before the study.

### 2.3. Inclusion and Exclusion Criteria

All the adult government employees of both genders and who participated in the health campaign during the study period in Bahrain were included in the sample. Pregnant women and cardiac patients were excluded from the sample. If a particular machine, such as the near patient testing device (LDX system for testing blood for lipids and the carbon monoxide monitor), is defaulted during a planned campaign, data was excluded from the analysis and the related data was considered missing.

### 2.4. Test Procedures and Measurement Tools

Data was collected by face-to-face interviews of participants by a comprehensively trained team and filling of standardized bilingual (Arabic and English) questionnaires. The team was composed of medical record staffs, nurses, laboratory technicians, doctors, a health promotion specialist, a driver, and a nutritionist. Structured interviews were conducted with the participated employees in designated rooms in each workplace during working hours. Standardized serially numbered forms for the participant employee data were used. The forms were composed of* demographic data section* including the campaign date, employee's name, central population registry (CPR), age, gender, nationality, marital status, educational level, occupation, primary local healthcare center, and contact number.* The second section* was composed of questions about behavioral risk factors for CVD such as smoking status, physical activity, fruits and vegetables intake and number of servings per day, and questions about the use of seat belt while driving. The third section included questions about any diagnosed NCD, treatment, and related family history, and any previous screening for cervical or breast cancer. The last section was composed of record of the basic physical and biochemical measurements including the participants' weight (Wt. in Kg), height (Ht. in cm), body mass index (BMI in Kg/m^2^), waist circumference (WC in cm), near patient testing of random blood glucose (RBG in mg/dL), (TC, LDL-C, HDL-C, TG in mmol/dL), and level of measured expired carbon monoxide (CO in ppm) for smokers (represents the percentage of carboxyhemoglobin). In addition, the last section had a summary of the composite CVD risk factors as defined by including current daily smoking, less than five servings of fruits and vegetables, low level of physical activity, overweight and obesity, and HTN. The final doctors' decision or advice was also included in the form. The roles of each member of the team were specified.

A well-calibrated sphygmomanometer and digital blood pressure machines were used for blood pressure measurements, measuring tapes for the WC, weighing scales for the waist circumference (WC), weighing scales for the Wt., HT., and BMI measurements. The Cholestech L.D.X System consists of a compact, portable,lightweight (<1 Kg) electronic analyzer and a series of unique, single-use disposable casettes. The L.D.X System delivers the ability to measure a complete lipid profile and glucose and it does it all in 5 minutes per test cassette. The system is evaluated by the UK medical devices Agency. Moreover, Carbon Monoxide (CO) measurements were taken for smokers using a device called a CO monitor.

For blood pressure measurement, participants' conditions were ensured to follow the BP measurement guidelines including* patient's posture*,* circumstances*,* cuff size*,* manometer calibration* every six months against a mercury manometer,* the technique*, and* blood pressure recordings*.

The equipment used for the cholesterol near patient testing includes single use safety lancets, cholesterol testing cassettes, septic wipes, sterile gauze, capillary tube to collect blood, gloves, hand wash, papers bin, and hypoallergenic Elastoplast.

A standardized procedure was as followed including wearing gloves, ensuring hand wash, using the participants' ring or index finger after warming it for the blood to flow better, wiping it with antiseptic, and drying it with gauze. The participant's finger then pricked after informing and reassuring. The first drop of blood was wiped away with gauze and a capillary tube was used to suck up blood from the finger (the participant was asked to hold the hand with the fingers pointing down to help with the blood flow). Sometimes the fingers have to be squeezed slightly to get the blood out, but not too much so that the quality of blood sample is not affected by contaminating it with the tissue fluids or skin cells. The blood drop was put into the cassette. When the test is conducted, participant's results were recorded on their questioners and used sharp materials, used L.D.X cassettes with the soiled materials were kept directly in sharp containers to be handled as per infection control guidelines. The machine needed to be regularly calibrated and quality assured by the providing company. Appropriate training was given on use of the machines and measurements tools in use. All users had Hepatitis B cover.

## 3. Ethical and Confidentiality Considerations

Ethical approvals, data protection, and confidentiality were anticipated and dealt with in a professional manner. However, there were no written consents as the study was from a health survey in which the employees participated after verbally being informed about the need for CVD risk factors data among employees. Ministry of health and a pharmaceutical company funded the health campaign. The funder from any pharmaceutical company was not included in the data analysis or collection.

## 4. Methods of Data Analysis

The data were entered and analyzed using descriptive and inferential statistics where appropriate. Descriptive statistics were used to summarize the data. Means and standard deviations were used to represent quantitative variables and percentages or proportions for categorical variables. In addition, the computer programs STATA and Excel were used to tabulate frequencies and produce graphs. The response rate was calculated and was around 99.9%.

## 5. The Results

The majority of the participated employees were Bahraini (93.9%). The mean age of the participants was 39.1 years, with 43.7% women and 56.3% men. The majority had graduated from colleges and 27.2% completed their basic education. Almost 80% of the participants were staff and 13.6% were in the management.

The participants' distribution according to some behavioural risk factors was demonstrated in Table 1 and related further analysis represented in Table 4. Only 16.1% of the participants in the campaign were using any type of tobacco during the study period. The carbon monoxide was measured in parts per million (ppm). In 60.7% of them the results were in the nonsmoker range (0–6 ppm) while 20.8% had low dependence (7–15 ppm) and 18.6% were strongly dependent (≥15).

Among 905 who responded to the physical activity questions, 50.8% were not physically active. Sufficient physical activity was defined as engaging in moderate-intensity physical activity or walk for at least 30 minutes 5 days per week or 20 minutes of vigorous activity 3 days/week.

Among 903 participants, 75.4% were eating fruits and vegetables as part of their diet. Among those, 81% were found to eat 1-2 servings of fruits and vegetables per day. Only 4.9% were found to be eating ≥ 5 servings per day of fruits and vegetables.

Eating ≥ 5 servings of fruit and vegetables are considered protective from cardiovascular event.

Table 2 represents some physical measurements of the participants and related further analysis is represented in Table 4. Among 1138 participants, 36.4% had their systolic blood pressure < 120 mmHg. Only 21.6% had their systolic blood pressure ≥ 140 mmHg. Among the 1138 participants, 44.2% had their diastolic blood pressure < 80 mmHg. 23.3% had blood pressure levels ≥ 90 mmHg.

The prevalence of reported hypertension among the participants was 36.9% that included the participants who were not on treatments and those who were on treatment. Further hypertension data analysis by gender is in Table 4.

**Figure 1:**
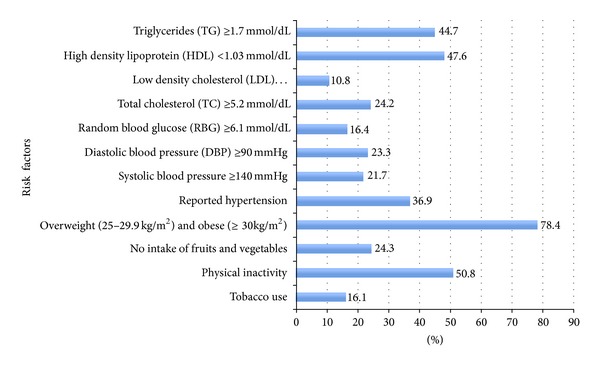
The prevalence of cardiovascular disease risk factors among the participants (employees).

The body mass index (BMI) measurements demonstrated that 39.7% of the participants were overweight (BMI 25–29.9 Kg/m²) and 38.7% were obese (BMI ≥ 30 Kg/m²). The overall prevalence of overweight and obesity among the participants is 78.4% as shown in Figure 1. The prevalence of the overweight and obesity by gender is described in the following section (see Table 4). The waist circumference measurements, which were taken for 395 female participants, demonstrated that 88.6% of them had a measurement of ≥80 cm. The waist circumference measurements for 495 male participants demonstrated that in 62.4% of them, it was ≥ 94 cm in males.

Table 3 demonstrates some biochemical measurements of the participants. Among the tested participants (1123), 44.7% had triglycerides levels of ≥ 1.7 mmol/dL.

The total cholesterol was tested in 1104 participants with 24.2% having levels ≥ 5.2 mmol/dL.

Among the 1007 tested participants, the majority (62.4%) had their LDL levels < 2.6 mmol/dL. 26.8% of them had it between 2.6 and 3.3 mmol/dL, while only 10.8% had their LDL levels above 3.3 mmol/dL.

Among the 1121 participants who were tested for HDL-C, 47.55% had their HDL levels ≤ 1.03 mmol/dL, while only 12.31% had it ≥ 1.55 mmol/dL.

Further HDL results levels analysed by gender are discussed in Table 4 with further analysis. The total number of participants who were tested for random blood glucose was 1113. Only 16.3% had levels of ≥6 mmol/dL.

**Figure 2:**
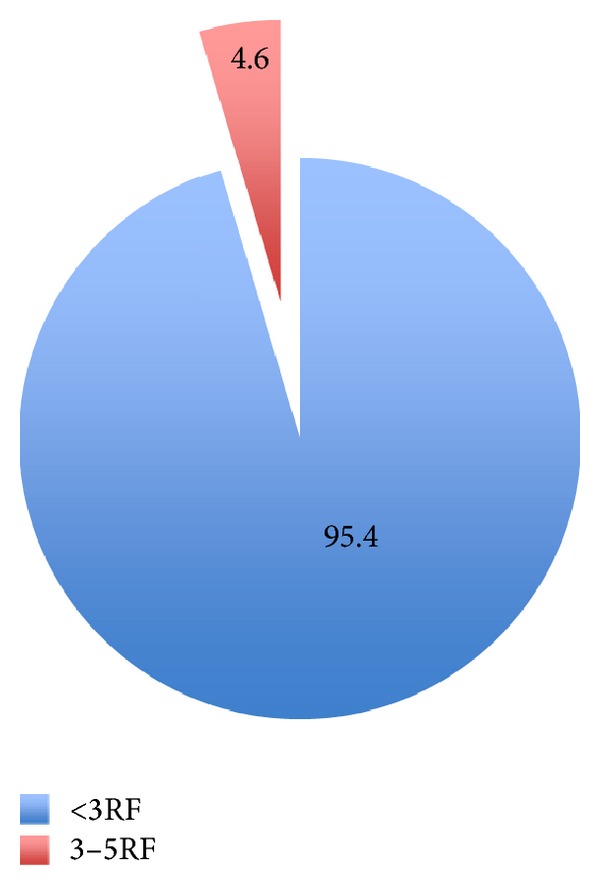
The distribution of composite cardiovascular disease risk factors among the participants (employees).

Figure 2 summarizes the cardiovascular composite risk factors and referrals among the participants. The majority of 1139 participated employees (95.4%) had either no or less than 3 cardiovascular risk factors while only (4.65%) had 3–5 risk factors. Among 1068 participants, the majority (85.5%) was referred to local health centre for further evaluation and follow-up. Referral was for preventing one or more of the composite risk factors through primary health care preventive services. Figure 1 represents the prevalence of cardiovascular disease risk factors among the participants.

### 5.1. Results of Further Analysis of Some of the CVD Risk Factors

As per Table 4 the overall prevalence of tobacco use (smoking) among the participants was 16.0%, with prevalence of 25.3% among males and of 4.0% among females with a significant difference in tobacco use among gender; 89% of the tobacco users were males and 10.9% were females as in Table 4 (*P* value < 0.001).

The overall prevalence of overweight among the participants was 39.8% with prevalence among the males of 42.9% and 23.6% among females. The overall prevalence of obesity among the participants was 38.7% with 36.97% prevalence among the males and 40.9% among females. The mean values of BMI between males and females were compared using the unpaired *t*-test. The mean BMI was 0.17 kg/m² higher among the females, but this difference was not statistically significant (*P* value < 0.6) (95% CI −0.83 to 0.49) as in Table 4.

The overall prevalence of physical inactivity among the participants was 50.8% with aprevalence of 60.6% among females and 43.3% among males. There was a significant difference in physical activity among gender, with 64.6% of the physically active being males, while only 35.3% of them were females (*P* value < 0.001).

The prevalence of reported hypertension among the participants was 36.9% and that included the participants who were on and not on treatments. The prevalence of hypertension based on systolic blood pressure readings is 21.7% and 23.4% based on diastolic blood pressure readings.

The prevalence of hypertension based on systolic blood pressure readings among males was 27.5% and 14.3% among females. The mean systolic blood pressure was 9.2 mmHg higher among the males, and this difference was statistically significant (*P* value < 0.001) (95% CI 7.2 to 11.2) as in Table 4.

The prevalence of hypertension based on diastolic blood pressure readings among males was 31.4% and 13.1% among females. The results show the mean diastolic blood pressure was 6.6 mmHg higher among the males, and this difference was statistically significant (*P* value < 0.001) (95% CI 5.3 to 7.9) as in Table 4.

The prevalence of low HDL level (<1.03 mmol/dL) among the male participants was 64.1% while it was 26.6% among the females. The results show the mean high-density lipoprotein was 0.3 mmol/L higher among the females, and this difference was statistically significant (*P* value < 0.001) (95% CI −0.3 to −0.23) as in Table 4.

## 6. The Discussion

There is an emerging increase in the prevalence of CVD risk factors in Middle East. Age-standardized death rates per 100000 population in Kingdom of Bahrain by gender and cause were 68.8 for Ischaemic Heart Disease (IHD) and 28.2 for cerebrovascular disease [1, 9]. Despite the high CVD related mortality and morbidity and established national plans, the CVD risk factors surveillance data are limited and epidemiological studies in this field from this region were mainly cross sectional and at individual country levels. This is another cross-sectional study of CVD risk factors among an important group of the community that described their characteristics to help in health promotion.

While conducting this study there were some problems met with deciding the inclusion and exclusion criteria. There were some difficulties in getting biochemical results of some participants due to high triglyceride levels, which lead to incomplete or missing data.

As data was collected from different workplaces, at intervals, there may be an issue with the reliability and consistency of the data based on the blood pressure, height, and weight measurements. Some information may be missing from the data sets or may have been erroneously entered. Moreover, since it is an observational study design, there was no control over the participants' characteristics with possible major sources of bias such as sampling, measurements, volunteering, and information. Analyzing the blood pressure and low-density lipoprotein levels data was difficult as the cutoff points were different across guidelines. Some variables did not show normally distributed. That needed some experience for the decision to report the mean or the interquartile range.

### 6.1. Physical Inactivity

Insufficient physical inactivity is associated with 20%–30% increased risk of all-cause mortality [10]. Participation in 150 minutes of physical activity of moderate intensity per week was estimated to reduce the Ischemic heart disease by around 30% and diabetes risk by 27% [11]. The study demonstrated that the majority of the participants were young, educated, and qualified employees, yet around half of them did not practice any type of physical activity. Moreover, the physical activity was insufficient among the majority of those who considered themselves physically active. Similar results about physical inactivity were demonstrated in a previous national survey [2] and a study of male university students in Dammam City, Saudi Arabia [7].

Numerous studies have demonstrated the association between the physical inactivity “westernization” (a change from traditional lifestyle, one that incorporates manufactured foods and sedentary employment) and obesity. Physical inactivity can lead to prediabetes, diabetes, hypertension, and dyslipidemia [12].

Although the employees recognize the overall health benefits of regular and sufficient physical activity, the CVD associated benefits are not well recognized. Moreover, the employers were not sufficiently promoting this healthy behavior among their employees during their working hours. The employees are not able to be physically active after their working hours because of their roles in the family or community. Similarly, a survey of NCD related risk factors of Iranian adults in 2007 demonstrated a linear association between the number of metabolic abnormalities and lower levels of physical activity [13]. The prevalence of physical inactivity in this study was more among the female participants, with similar findings from a previous national survey [2].

### 6.2. Tobacco Use (Smoking)

Smoking is a very common avoidable risk factor for CVD. There are about one billion current smokers in the world, and the prevalence of daily smoking varied across WHO regions [10]. The highest overall prevalence is estimated to be 31% in the WHO European region [14]. The prevalence of current tobacco use (smoking) among the employees in this study was almost similar to overall prevalence and gender distribution of smoking revealed in the national survey of NCD risk factors [2] and in a systematic review from the Middle East region [15].

### 6.3. Fruits and Vegetables Intake

Low fruits and vegetables intake is one of the dietary behaviors that were linked with increased risk for CVD (Mendis et al., 2010). Although this study demonstrated quite high intake of fruits and vegetables among the participants, only a very small percentage was taking more than 5 servings per day that is considered cardioprotective from the trials. Similar findings were reported from the national survey. According to Gaziano, the dietary pattern changes in the Middle East were shifted from traditional fiber rich and low fat into low fiber, high sugar content with decline in fruits and vegetables intake and high fat. The fast and preserved foods are more abundant and the fat intake has risen [16, chapter 33].

The oil rich countries such as Kuwait, Qatar, Saudi Arabia, United Arab Emirates, Bahrain, Oman, and Yemen are characterized by high intakes of red meat, carbohydrates, and sugar [17].

### 6.4. Overweight, Obesity, and Central Obesity

Obesity prevalence is rising in the world. It was associated with the major CVD risk factors and has adverse metabolic effects on lipids, blood glucose, and blood pressure [18]. The overall prevalence of overweight and obesity and their distribution among genders were almost equal to the overall prevalence in the national survey [2] and also similar to results from the international day for the evaluation of abdominal obesity (IDEA) [19].

The prevalence of obesity in the Middle East as a whole was found to be higher in women than in men, but the rates of obesity were similar in both sexes [15].

In Isfahan Healthy Heart program, 3694 participants were measured. There was a 35.9% prevalence of overweight among women and 28.1% obesity [3].

This study revealed 62.4% of males to have abdominal obesity (WC ≥ 94 cm) and 88.6% of females (WC ≥ 80 cm). Although studies using waist circumference as an indicator or measure of obesity in this region are scanty, the high prevalence of central obesity in both genders is understandable with the overall high prevalence of obesity and physical inactivity. The prevalence of metabolic syndrome was 19.8% in women and 63% in obese women. In men, corresponding values were 3.7%, 18.0%, and 40.1% as was demonstrated by [20].

### 6.5. Hypertension

Hypertension is a major CVD risk factor. This study showed similar overall hypertension prevalence and gender distribution to the national survey [2], with it being higher among the male participants compared to its prevalence among the females and higher than that of history of diagnosed hypertension from the survey [2] as it is self-reported and overlap of diagnosed cases can be expected. The results of this study revealed higher hypertension prevalence than a study done in Saudi Arabia among university students (Amr et al., 2007), and the reason in the difference could be due to younger age group in the Saudi study (Amr et al., 2007) or the reporting of different cutoff levels for the blood pressure between the two studies.

The hypertension prevalence from this study was about the same as other surveys in Iranian [13, 21].

### 6.6. Diabetes

The study showed that the random blood glucose was ≥ 6.1 mmol/dL is almost the same prevalence of diabetes that was demonstrated in the national survey [2]. Although diabetes mellitus is usually diagnosed by measuring the fasting blood glucose of ≥7 mmol/dL, the random blood glucose of ≥6.1 mmol/dL was chosen as a cutoff in this study as the study was done during a working day, and the participants were targeted for early detection and intervention of CVD risk factors. The rates of diabetes in the Middle East were demonstrated to be the highest [22]. Esteghamati et al. in the third national surveillance of NCD risk factors in Iran demonstrated a prevalence of diabetes of 8.7% [13].

### 6.7. Dyslipidemia

Dyslipidemia (high LDL, low HDL, and high TG) is associated with increased CVD risk (WHO, 2010). The study showed the prevalence of total cholesterol level of ≥5.2 mmol/dL to be much lower than that found in the national survey (40.6%) [2] and from Iran third national survey [13].

The study showed the LDL levels among the participants to be mainly in <2.6 mmol/dL (62.36%), but in 26.81% of them it was in the range of 2.6–3.3 mmol/dL. The high prevalence of lipids disorders was demonstrated in studies [21].

HDL was < 1.03 mmol/dL among high percentage of the participants which along with unhealthy behavior of physical inactivity and unhealthy diet, obesity, and other dyslipidemias can contribute to CVD risk. Similar findings of a low HDL prevalence of have been found in Iran [23, 24].

This study found high triglycerides levels of the participants, almost the same as those found in Iran third national survey [13].

### 6.8. Composite CVD Risk Factors

Only (4.65%) had 3–5 risk factors. A similar finding was found in a survey from Oman [25].

## 7. The Conclusion

In conclusion, CVD risk factors have high prevalence in the Middle East region including behavioural, physical, and biochemical measurements.

Despite high prevalence of individual CVD modifiable and major risk factors, the prevalence among the participants of composite CVD risk factors was found to be low. That can encourage for the recommendation for interventions and lifestyle modifications at the population level and at work sites.

The findings of the analysis were in line with many studies conducted at the country level and in the region.

The high prevalence of CVD risk factors among participated employees reflected alarming public health concerns and a future health demand. It constitutes a threat if health promotion and awareness programs are not well designed.

Although NCD indicators are set, health policies are in place, clinical guidelines for major CVD risk factors are available, continuous surveillance for CVD risk factors are to be strengthened, and guidelines for CVD detection and prevention are needed. Moreover, future researches are recommended.

## Figures and Tables

**Table 1:** The behavioural risk factors of the participants (employees).

Number	Characters	Males	Females	Total number (%)
1	Tobacco use			**1137**
	Yes	**162**	**20**	182 (**16.1)**
	No	**479**	**476**	955 (**83.9)**

2	Type of tobacco			**183**
	Cigarette			111 (**60.7**)
	Shisha			43 (**14.8**)
	Cigarette and shisha			27 (**23.5**)
	Not asked			2 (0.6)

3	Carbon monoxide (CO) measurements among smokers (ppm)			**183**
	0–6 nonsmoker			111 (**60.7**)
	7–15 low dependence			38 (**20.8**)
	>15 strongly dependent			34 (**18.6**)

4	Physical activity			**904**
	Yes	287	**157**	444 (**49.2**)
	No	219	**241**	460 (**50.8**)

5	Physical activity frequency∗			**890**
	Yes			320 (**35.9**)
	No			520 (**58.4**)
	Not asked			3 (0.3)
	Not applicable			47 (5.3)

6	Fruits and vegetables intake			**903**
	Yes			683 (75.4)
	No			220 (24.3)

7	Fruits and vegetables servings per day∗∗			**678**
	1-2/day			549 (81)
	3-4/day			94 (13.9)
	≥5/day			33 (4.9)
	Not asked			2 (0.2)

*Sufficient physical activity was defined as engaging in moderate-intensity physical activity or walking for at least 30 minutes 5 days per week or 20 minutes of vigorous activity 3 days/week.

**Eating ≥5 servings of fruit and vegetables is considered protective from cardiovascular event.

**Table 2:** The physical measurements of the participants (employees).

Number	Characters	Males	Females	Total number (%)
1	Body mass index (BMI) in Kg/m^2^			**1137**
	Underweight (<18.5 Kg/m^2^)	**4**	**6**	10 (0.9)
	Normal weight (18.5–24.9 Kg/m^2^)	**125**	**110**	235 (**20.7**)
	Overweight (25–29.9 Kg/m^2^)	**275**	**117**	452 (**39.7**)
	Obese (≥30 Kg/m^2^)	**237**	**203**	440 (**38.7**)

2	Waist circumference (males)		**Not applicable**	**495**
	<94 cm	186		186 (37.6)
	≥94 cm	309		309 (**62.4**)

3	Waist circumference (females)	**Not applicable**		**395**
	<80 cm		45	45 (11.4)
	≥80 cm		350	350 (**88.6**)

4	Systolic blood pressure (sbp)			**1137**
	<120 mmHg	**166**	**247**	413 (**36.4**)
	120–139 mmHg	**298**	**179**	477 (**41.9**)
	≥140 mmHg	**176**	**71**	247 (**21.7**)

5	Diastolic blood pressure (dbp)			**1137**
	<80 mmHg	**214**	**288**	502 (44.2)
	80–89 mmHg	**225**	**144**	369 (32.5)
	≥90 mmHg	**201**	**65**	266 (**23.3**)

**Table 3:** The biochemical measurements of the participants (employees).

Number	Characters	Males	Females	Total number (%)	Number
1	Random blood glucose (RBG)			**1112**	**5.8 (SD 2.5)**
	≤6 mmol/dL	**502**	**428**	930 (**83.7**)
	≥6.1 mmol/dL	**121**	**61**	182 (**16.4**)

2	Total cholesterol (TC)			**1103**	**4.7 (SD 0.9)**
	≤5.1 mmol/dL	**447**	**389**	836 (**75.8**)
	≥5.2 mmol/dL	**171**	**96**	267 (**24.2**)

3	Low density cholesterol (LDL)			**1006**	**2.8 (SD 0.8)**
	<2.6 mmol/dL	**321**	**307**	628 (**62.4**)
	2.6–3.3 mmol/dL	**167**	**102**	269 (**26.8**)
	3.36–4.1 mmol/dL	**55**	**37**	92 (**9.1**)
	4.14–4.89 mmol/dL	**9**	**5**	14 (1.4)
	≥4.92 mmol/dL	**2**	**1**	3 (0.3)

4	High density lipoprotein (HDL)			**1120** (%)	**1.1 (SD 0.4)**
	<1.03 mmol/dL	400 (64.1)	**132** (26.6)	532 (**47.6**)
	1.03–1.54 mmol/dL	184 (29.5)	266 (53.6)	450 (**40.1**)
	≥1.55 mmol/dL	40 (6.4)	98 (19.8)	138 (**12.3**)

5	Triglycerides (TG)			**1123**	**1.9 (SD 1.2)**
	≤1.69 mmol/dL			621 (**55.3**)
	≥1.7 mmol/dL			502 (**44.7**)

**Table 4:** Comparison of some risk factors between genders among the participants.

Number	Variable	Male number (%)/Mean (SD)	Female number (%)	Total/(95% CI)	*P* value
1	Tobacco use	162 (89)	20 (10.99)	182 (100)	0.001∗

2	BMI	Mean 29.1 (5.3)	Mean 29.3 (5.98)	The mean BMI was 0.17 kg/m^2^ higher among females 0.2 (−0.8 to 0.49)	<0.6∗∗

3	Physical inactivity	219 (47.6)	241 (52.4)	460 (100)	0.001∗

4	Systolic blood pressure (sbp)	127.4 (17.1)	118.1 (17.1)	The mean sbp was 9.2 mmHg higher among males 9.2 (7.2 to 11.2)	<0.001∗∗

5	Diastolic blood pressure (dbp)	80.9 (11.1)	74.3 (11.5)	The results show the mean dbp was 6.6 mmHg higher among the males 6.6 (5.3 to 7.9)	<0.001∗∗

6	High density lipoprotein (hdl)	0.98 (0.41)	1.3 (0.4)	The mean high density lipoprotein was 0.3 mmol/L higher among the females 0.32 (−0.3 to −0.23)	<0.001∗∗

*The Chi-square test was used.

**The unpaired *t*-test was used.

## References

[1] (2011). *Global Atlas on Cardiovascular Disease Prevention and Control*.

[2] (2010). *National Non-Communicable Health Survey*.

[3] Kelishadi R, Gharipour M, Sadri GH, Tavasoli AA, Amani A (2008). Cardiovascular disease risk factors, metabolic syndrome and obesity in an Iranian population. *Eastern Mediterranean Health Journal*.

[4] Al-Assomi F, Al-Kandari S, Al-Wadaani D, Thalib L (2005). Prevalence of cardiovascular risk factors amongst the population of Surra, Kuwait. *Journal of the Bahrain Medical Society*.

[5] Baynouna LM, Revel AD, Nagelkerke NJ (2008). High prevalence of the cardiovascular risk factors in Al-Ain, United Arab Emirates. An emerging health care priority. *Saudi Medical Journal*.

[6] Hajat C, Harrison O, Al Siksek Z (2012). Weqaya: a population-wide cardiovascular screening program in Abu Dhabi, United Arab Emirates. *The American Journal of Public Health*.

[7] Sabra AA, Taha AZ, Al-Sebiany AM, Al-Kurashi NY, Al-Zubier AG (2007). Coronary heart disease risk factors: prevalence and behavior among male university students in Dammam City, Saudi Arabia.. *The Journal of the Egyptian Public Health Association*.

[8] Al-Hazzaa HM (2004). Prevalence of physical inactivity in Saudi Arabia: a brief review. *Eastern Mediterranean Health Journal*.

[9] Mendis S, Puska P, Norrving B (2011). *Global Atlas on Cardiovascular Disease Prevention and Control*.

[10] Mendis S, Lindholm LH, Mancia G (2007). World Health Organization (WHO) and International Society of Hypertension (ISH) risk prediction charts: assessment of cardiovascular risk for prevention and control of cardiovascular disease in low and middle-income countries. *Journal of Hypertension*.

[11] UNAIDS (2007). *Prevention of Cardiovascular Disease*.

[12] Zindah M, Belbeisi A, Walke H, Mokdad AH (2008). Obesity and diabetes in Jordan: findings from the behavioral risk factor surveillance system, 2004. *Preventing Chronic Disease*.

[13] Esteghamati A, Khalilzadeh O, Ashraf H (2010). Physical activity is correlated with serum leptin independent of obesity: results of the national surveillance of risk factors of noncommunicable diseases in Iran (SuRFNCD-2007). *Metabolism*.

[14] World Health Organization (2011). *WHO Report on the Global Tobacco Epidemic, 2011. Warning about the Dangers of Tobacco*.

[15] Motlagh B, O'Donnell M, Yusuf S (2009). Prevalence of cardiovascular risk factors in the middle east: a systematic review. *European Journal of Cardiovascular Prevention and Rehabilitation*.

[16] Measham AR, Alleyne G, Mills A (2006). *Disease Control Priorities in Developing Countries*.

[17] Shara NM (2010). Cardiovascular disease in Middle Eastern women. *Nutrition, Metabolism and Cardiovascular Diseases*.

[18] Alwan A (2011). *Global Status Report on Noncommunicable Diseases 2010*.

[19] Balkau B, Deanfield JE, Després J (2007). International day for the evaluation of abdominal obesity (IDEA): a study of waist circumference, cardiovascular disease, and diabetes mellitus in 168 000 primary care patients in 63 countries. *Circulation*.

[20] Yusuf S, Reddy S, Ôunpuu S, Anand S (2001). Global burden of cardiovascular diseases. Part I: general considerations, the epidemiologic transition, risk factors, and impact of urbanization. *Circulation*.

[21] Janghorbani M, Amini M, Gouya MM, Delavari A, Alikhani S, Mahdavi A (2008). Nationwide survey of prevalence and risk factors of prehypertension and hypertension in Iranian adults. *Journal of Hypertension*.

[22] Harati H, Hadaegh F, Saadat N, Azizi F (2009). Population-based incidence of Type 2 diabetes and its associated risk factors: results from a six-year cohort study in Iran. *BMC Public Health*.

[23] Sharifi F, Mousavinasab SN, Soruri R, Saeini M, Dinmohammadi M (2008). High prevalence of low high-density lipoprotein cholesterol concentrations and other dyslipidemic phenotypes in an Iranian population. *Metabolic Syndrome and Related Disorders*.

[24] Ebrahimi M, Kazemi-Bajestani S, Ghayour-Mobarhan M, Ferns G (2011). Coronary artery disease and its risk factors status in Iran: a review. *Iranian Red Crescent Medical Journal*.

[25] Al Riyami AA, Afifi M (2003). Clustering of cardiovascular risk factors among Omani adults. *Eastern Mediterranean Health Journal*.

